# Unraveling the mechanism of thermotolerance by Set302 in *Cryptococcus neoformans*

**DOI:** 10.1128/spectrum.04202-23

**Published:** 2024-06-14

**Authors:** Yue Ni, Yue Qiao, Xing Tian, Hailong Li, Yang Meng, Chao Li, Wei Du, Tianshu Sun, Keting Zhu, Wei Huang, He Yan, Jia Li, Renjie Zhou, Chen Ding, Xindi Gao

**Affiliations:** 1 College of Life and Health Sciences, Northeastern University, Shenyang, Liaoning, China; 2 Department of Emergency, Xinqiao Hospital, Army Medical University, Chongqing, China; 3 NHC Key Laboratory of AIDS Immunology, National Clinical Research Center for Laboratory Medicine, The First Affiliated Hospital of China Medical University, Shenyang, China; 4 Department of Clinical Laboratory, National Clinical Research Center for Laboratory Medicine, The First Affiliated Hospital of China Medical University, Shenyang, Liaoning, China; 5 Beijing Key Laboratory for Mechanisms Research and Precision Diagnosis of Invasive Fungal Diseases, Beijing, China; 6 Medical Research Centre, State Key Laboratory of Complex Severe and Rare Diseases, Peking Union Medical College Hospital, Chinese Academy of Medical Science, Beijing, China; University of Minnesota Medical School, Minneapolis, Minnesota, USA

**Keywords:** thermotolerance, *Cryptococcus neoformans*, misfolded protein

## Abstract

The underlying mechanism of thermotolerance, which is a key virulence factor essential for pathogenic fungi such as *Cryptococcus neoformans*, is largely unexplored. In this study, our findings suggest that Set302, a homolog of Set3 and a subunit of histone deacetylase complex Set3C, contributes to thermotolerance in *C. neoformans*. Specifically, the deletion of the predicted Set3C core subunit, Set302, resulted in further reduction in the growth of *C. neoformans* at 39°C, and survival of transient incubation at 50°C. Transcriptomics analysis revealed that the expression levels of numerous heat stress-responsive genes altered at both 30°C and 39°C due to the lack of Set302. Notably, at 39°C, the absence of Set302 led to the downregulation of gene expression related to the ubiquitin-proteasome system (UPS). Based on the GFP-α-synuclein overexpression model to characterize misfolded proteins, we observed a pronounced accumulation of misfolded GFP-α-synuclein at 39°C, consequently inhibiting *C. neoformans* thermotolerance. Furthermore, the loss of Set302 exacerbated the accumulation of misfolded GFP-α-synuclein during heat stress. Interestingly, the *set302∆* strain exhibited a similar phenotype under proteasome stress as it did at 39°C. Moreover, the absence of Set302 led to reduced production of capsule and melanin. *set302∆* strain also displayed significantly reduced pathogenicity and colonization ability compared to the wild-type strain in the murine infection model. Collectively, our findings suggest that Set302 modulates thermotolerance by affecting the degradation of misfolded proteins and multiple virulence factors to mediate the pathogenicity of *C. neoformans*.

**IMPORTANCE:**

*Cryptococcus neoformans* is a pathogenic fungus that poses a potential and significant threat to public health. Thermotolerance plays a crucial role in the wide distribution in natural environments and host colonization of this fungus. Herein, Set302, a critical core subunit for the integrity of histone deacetylase complex Set3C and widely distributed in various fungi and mammals, governs thermotolerance and affects survival at extreme temperatures as well as the formation of capsule and melanin in *C. neoformans*. Additionally, Set302 participates in regulating the expression of multiple genes associated with the ubiquitin-proteasome system (UPS). By eliminating misfolded proteins under heat stress, Set302 significantly contributes to the thermotolerance of *C. neoformans*. Moreover, Set302 regulates the pathogenicity and colonization ability of *C. neoformans* in a murine model. Overall, this study provides new insight into the mechanism of thermotolerance in *C. neoformans*.

## INTRODUCTION

For pathogenic fungi, thermotolerance is a major ecological trait, which has pre-adapted them to human pathogenicity by protecting them from heat stress in the natural and the host environment (1–5). As most fungi are less thermotolerant and the high temperature of the host is likely to act as a barrier to their growth (3, 6–8), growing evidence suggests that increasing global temperature will inadvertently select for thermotolerant fungi that are more likely to cause opportunistic human disease. A recent evolutionary ecological study of over 1,200 fungal species has shown a strong relationship between opportunistic pathogenicity and thermotolerance (9), and more lineages of opportunistic human pathogens intend to emerge from more thermotolerant fungi (10, 11). Notably, the global outbreak of the new heat-resistant pathogenic fungus *Candida auris* has attracted attention because of its high-temperature resistance, and *C. auris* is hypothesized to have arisen from high environmental temperatures (12, 13).

*Cryptococcus neoformans* is an environmental basidiomycete yeast causing over 180,000 deaths annually worldwide (14, 15). Its distribution in tropical areas and survival in pigeons are closely related to thermotolerance (16). Cryptococcus consists of 37 species, and the most common species associated with human disease are *C. neoformans*, *Cryptococcus deneoformans*, and *Cryptococcus gattii* (17–19). Some species, such as *Cryptococcus albidus*, *Cryptococcus laurentii, Cryptococcus liquefaciens,* and *Cryptococcus amylolentus*, are less virulent due to their temperature sensitivity (20, 21). Interestingly, a recent study showed that the incidence of cryptococcal meningitis is higher in hot environments, suggesting that heat stress may domesticate *C. neoformans* with a stronger infection ability (22). The importance of thermotolerance has been emphasized in various studies on the molecular mechanisms of *C. neoformans*, including membrane fluidity and cross-talk between unfolded protein response (UPR) and heat shock response (23–27). A recent study showed that the *C. neoformans CEL1* gene, encoding a lytic polysaccharide monooxygenase, affects thermotolerance and pathogenicity (28). Our recent research has revealed the role of cryptococcal Hsf3 in the defense of heat-induced reactive oxygen species (ROS) damage by modulating mitochondrial function (29). Thus, multiple pathways are utilized by *C. neoformans* to protect against heat stress, and the study of fungal heat adaptation mechanisms will help us to understand how heat-resistant fungi such as *C. neoformans* emerge and prevent more heat-resistant cryptococcal infections in the future.

Histone deacetylase complex Set3C is a conserved complex from yeast to mammals (30, 31). Set3 is the core and integral subunit, with two known domains: SET [Su (var)3-9, Enhancer-of-zeste and Trithorax] and PHD (32–35), which are essential for Set3 function. Set3C-mediated histone deacetylation is involved in various biological processes in *S. cerevisiae*, including meiosis, DNA repair, and stress response (32, 36–40). Set3C is required for infectious growth, conidiogenesis, and colonization in plant fungal pathogens (41, 42). Set3C complex is a key regulator of morphology transition and virulence in *Candida albicans* (43, 44). Set302 has been reported to affect yeast-hypha transition in *C. neoformans* (45). However, the role and mechanism of Set3C in the pathogenicity of *C. neoformans* is poorly understood.

Herein, we characterize the role of the predicted core subunit Set302 of histone deacetylase complex Set3C in the thermotolerance of *C. neoformans*. We discovered that Set302 deficiency led to reduced thermotolerance and viability at extreme temperatures, and the expression of ubiquitin-proteasome system (UPS) genes was positively regulated by Set302 and heat. Moreover, Set302 likely reduced the formation of misfolded proteins at high temperatures and performed the same function under proteasome stress. Furthermore, Set302 plays a role in regulating the pathogenicity to host. Overall, our findings emphasize the importance of Set302 in heat resistance and pathogenicity of *C. neoformans*.

## RESULTS

### Set3 is widely distributed and conserved in various fungi

**Fig 1:**
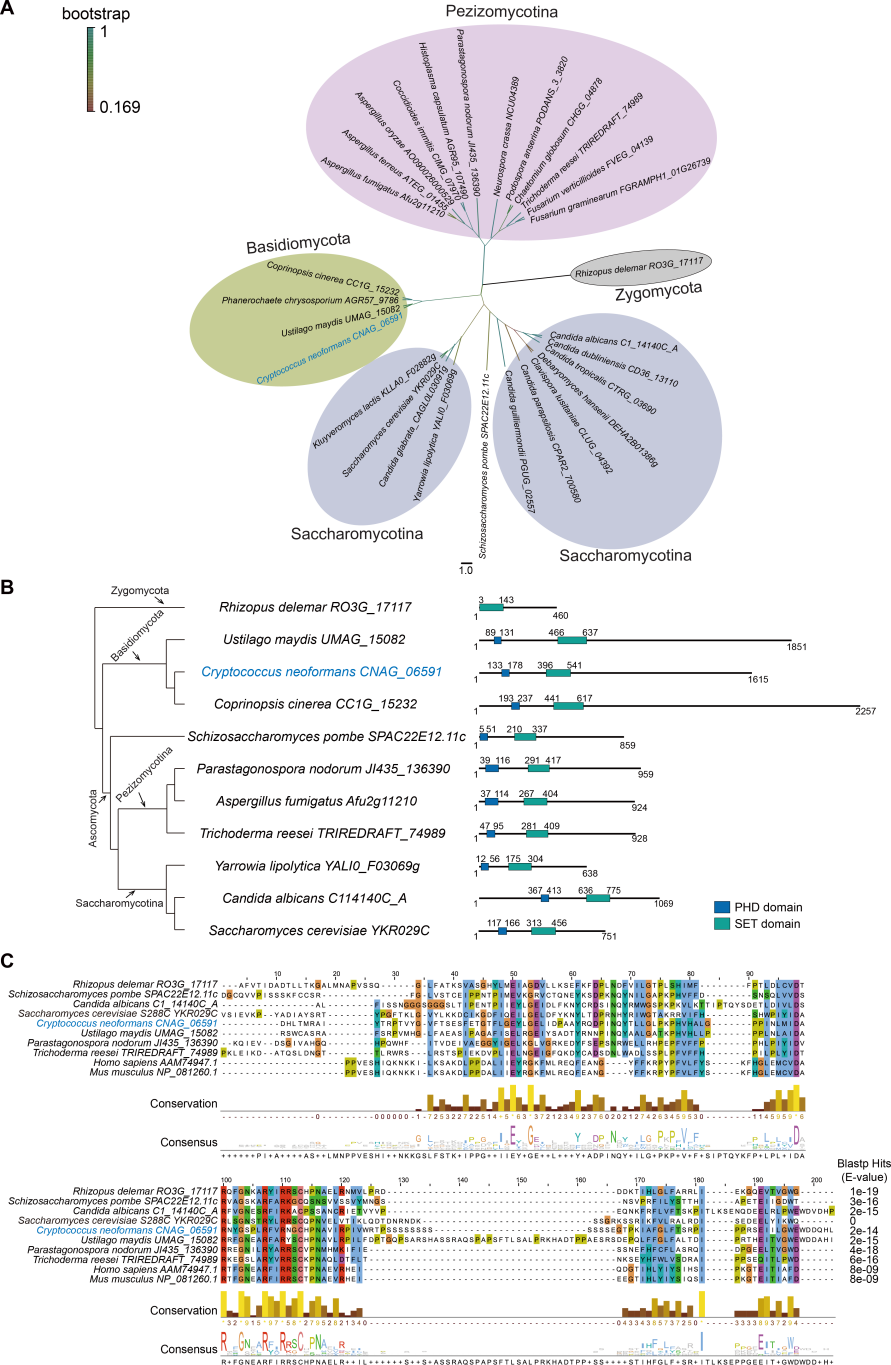
Set3 is broadly distributed and conserved across diverse fungi phyla. (**A**) Phylogenetic tree of Set3 amino acid sequences of representative fungi in distinct phyla. The tree was constructed using a neighbor-joining algorithm by MEGA5 and displayed using Figtree software. (**B**) The characterization of PHD and SET domain of Set3 protein from representative fungi. The domains were predicted using InterPro (http://www.ebi.ac.uk/interpro/). The blue and green boxes indicate the PHD and SET domains, respectively. The numbers represent the position of amino acids. (**C**) Multiple sequence alignment of SET domains in Set3 proteins. The SET domain sequences of Set3 proteins from the representative fungi from different phyla and mammals were used for alignment by Clustal Omega. The E-value was calculated by NCBI Blastp using the SET domain sequence of *S.cerevisiae* Set3 (YKR029C) as the template. Jalview software was employed for the calculation of conservation sites.

Set3C is a histone deacetylase complex, and Set3 is the core subunit for the structural integrity of the complex (31). To probe the distribution of Set3 in fungi, we surveyed 29 Set3 homologous proteins from different fungal phyla. The phylogenetic tree analysis showed that Set3 existed in multiple fungal phyla and that Set3 proteins of the same phylum tended to cluster in an identical group (Fig. 1A). The characterization of Set3 proteins from different fungi showed that, apart from *Rhizopus delemar* in the Zygomycota clade, Set3 homologous proteins in Basidiomycota, Pezizomycotina, and Saccharomycotina contain both the PHD finger and SET domains. Notably, Set3 amino acid sequences of Basidiomycota are much longer than those of other fungal phyla (Fig. 1B). Additionally, we compared the SET domain sequences from representative fungi and mammals and found that all SET domains had a significant E-value of BLASTP hits (E < 1e−6) compared to *S. cerevisiae* Set3, and there were multiple partially and totally conserved sites (Fig. 1C). Taken together, these results indicate that Set3 is widely distributed in different fungi and its key functional SET domain is well-conserved.

### The lack of Set302 results in decreased thermotolerance in *C. neoformans*

**Fig 2:**
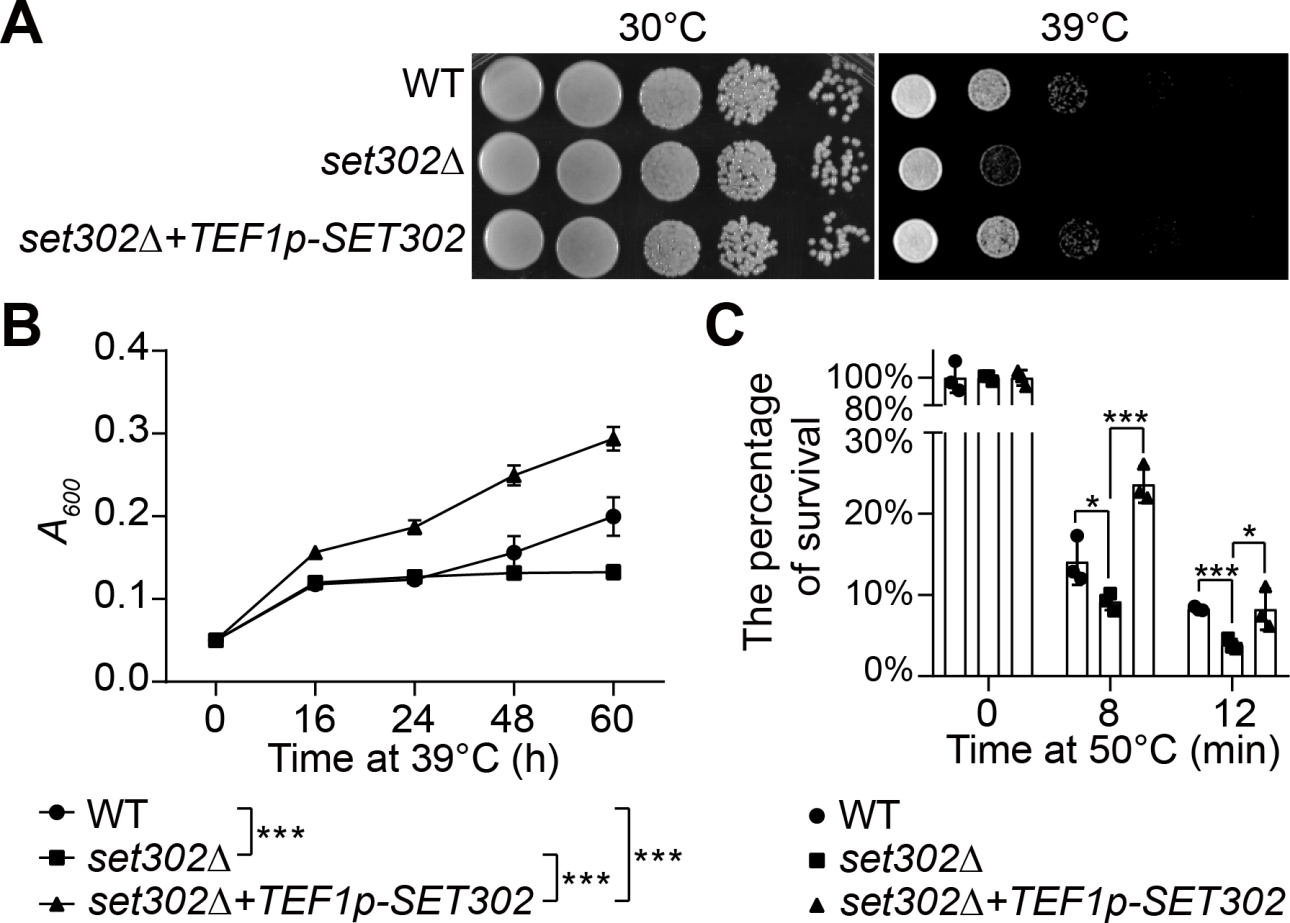
Set302 deficiency results in reduced thermotolerance of *C. neoformans*. (**A**) Spot dilution assays with wild-type (H99), *set302∆,* and *set302∆+TEF1p-SET302* strains were performed on YPD agar and incubated at the indicated temperature for 2 days. (**B**) Growth curve of fungal cells in liquid cultures. Wild-type, *set302∆,* and *set302∆+TEF1p-SET302* strains were grown in YPD liquid media at 39°C. Cell growths (absorbance at 600 nm) were measured at indicated time points. (**C**) The viability assay of wild-type, *set302∆,* and *set302∆+TEF1p-SET302* strains. The strains were plated onto YPD agar after the pretreatment at an extreme temperature of 50°C for the indicated time and incubated for 2 days at 30°C. CFUs were counted and normalized by the CFU of wild-type without heat treatment.

Although Set3 is critical in regulating the stress genes in *S. cerevisiae* (39), no evidence indicates that it contributes to thermotolerance in pathogenic fungi. To examine the role of the Set3 homologous protein Set302 of *C. neoformans* in heat resistance, we constructed the *set302∆* and *set302∆+TEF1p-SET302* strains. The results showed that *set302∆* strain exhibited normal growth at 30°C (Fig. 2A; Fig. S1A) and 37°C (Fig. S1B) but displayed a growth defect on YPD agar plate at 39°C (Fig. 2A). The reduced thermotolerance of *set302∆* strain was also observed in YPD liquid medium (Fig. 2B). This growth defect was cured by re-integrating the *SET302* gene controlled by the constitutive *TEF1* promoter (Fig. 2A and B). Interestingly, this *SET302* overexpression strain grew better than wild-type when incubated in liquid YPD medium at 39°C (Fig. 2B). Additionally, when subjected to 50°C for up to 12 minutes, *set302∆* strain exhibited reduced viability compared to wild-type, and *SET302* overexpression rescued the thermotolerance defect of *set302∆* strain (Fig. 2C). These results suggest that Set302 plays a role in thermotolerance by maintaining survival under heat stress.

### Transcriptome profiles governed by Set302 and heat stress

UPR and heat shock proteins (HSPs) are key for thermotolerance in fungi and mammals (25, 27, 46). The assay of phenotypes regulated by the UPR pathway, including growing ability with DTT treatment as endoplasmic reticulum stress and the integrity of cell membrane as detected by SDS, revealed no differences in growing ability between wild-type and *set302∆* strains (Fig. S2A). We also examined the phenotype regulated by the calcineurin pathway, which is important for the growth of *C. neoformans* under heat stress (25). However, we found no growth defect in the *set302∆* strain under calcium chloride (Fig. S2A) or cyclosporin A treatments (Fig. S2B). Additionally, the lack of *SET302* did not affect the HSP gene expression levels (Fig. S2C). Cryptococcal heat shock factor 3 (*Cn*Hsf3) was discovered as a regulator of thermotolerance by inhibiting mitochondrial ROS overload under heat stress (29). However, Set302 did not regulate *CnHSF3* gene expression (Fig. S2D). These results suggest that Set302 affects the thermotolerance of *C. neoformans* independently of these known pathways.

**Fig 3:**
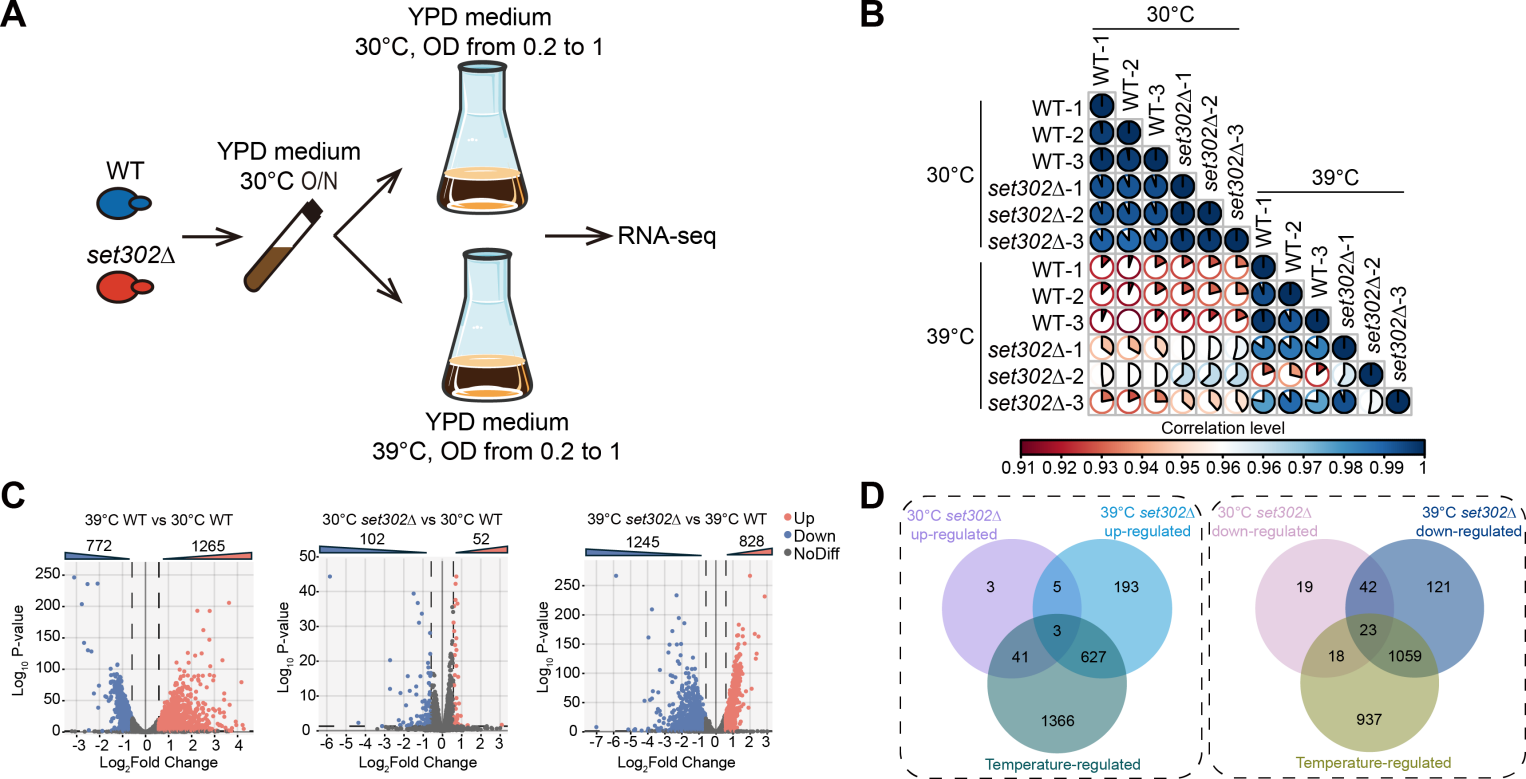
Transcriptome profiles governed by Set302 and heat stress. (**A**) Transcriptomics schematics for wild-type and *set302∆* strains. Wild-type and *set302∆* strains were grown in YPD medium overnight at 30°C and subcultured in fresh YPD medium with an OD_600_ of 0.2 until the OD_600_ reached 1 at 30°C or 39°C, respectively, for RNA-seq. (**B**) The correlation analysis of gene expression patterns between wild-type and *set302∆* strains from RNA-seq. The corrplot package was used to conduct the correlation analysis. (**C**) Volcano plot of differentially expressed genes between different strains at 30°C or 39°C. (**D**) Venn diagrams depict the comparison between the number of differentially expressed genes among the different groups in Fig. 3C.

To elucidate how Set302 contributes to thermotolerance, we performed an RNA-seq analysis of the wild-type and *set302∆* strains at 30°C and 39°C (Fig. 3A). We found that the correlation of gene expression between normal and high temperatures in the wild-type strain was low, indicating a significant switch in the gene expression pattern during temperature shift. Interestingly, we observed that the correlation of gene expression between wild-type and *set302∆* strains at high temperatures was lower than that under normal conditions, suggesting a more crucial role for Set302 under heat stress (Fig. 3B).

Next, we performed a detailed analysis of differentially expressed genes to identify the genes regulated by temperature and Set302. The RNA-seq data revealed that the expression of many genes changed from 30°C to 39°C, including 1,265 up-regulated genes and 772 down-regulated genes. A total of 154 genes showed altered expression at 30°C in the absence of *SET302*, including 102 down-regulated genes and 52 up-regulated genes. Intriguingly, the expression of 2,073 genes changed at 39°C due to the lack of *SET302*, consisting of 1,245 down-regulated genes and 828 up-regulated genes (Fig. 3C; Table S1). As the *set302∆* strain only showed defective growth at 39°C, we aimed to identify the genes responsive to heat stress and dependent on Set302 only at 39°C, but not at 30°C. The results showed that only 8 and 65 identical genes were up- and down-regulated by the *SET302* deficiency at 30°C and 39°C, respectively, indicating that Set302 has different functions at 30°C and 39°C. Furthermore, 627 genes responsive to heat stress were up-regulated and 1,059 genes responsive to heat stress were down-regulated in *set302∆* strain at 39°C, but not at 30°C (Fig. 3D). We speculated that the altered expression of these genes (627 and 1,059) may contribute to the defective thermotolerance of *set302∆* strain. We found no effect of Set302 on the gene expression of the known thermotolerance pathway in *C. neoformans* after comparing thermotolerance-related genes from CryptoNet with the differentially expressed genes between wild-type and *set302∆* strains (Fig. S3).

**Fig 4:**
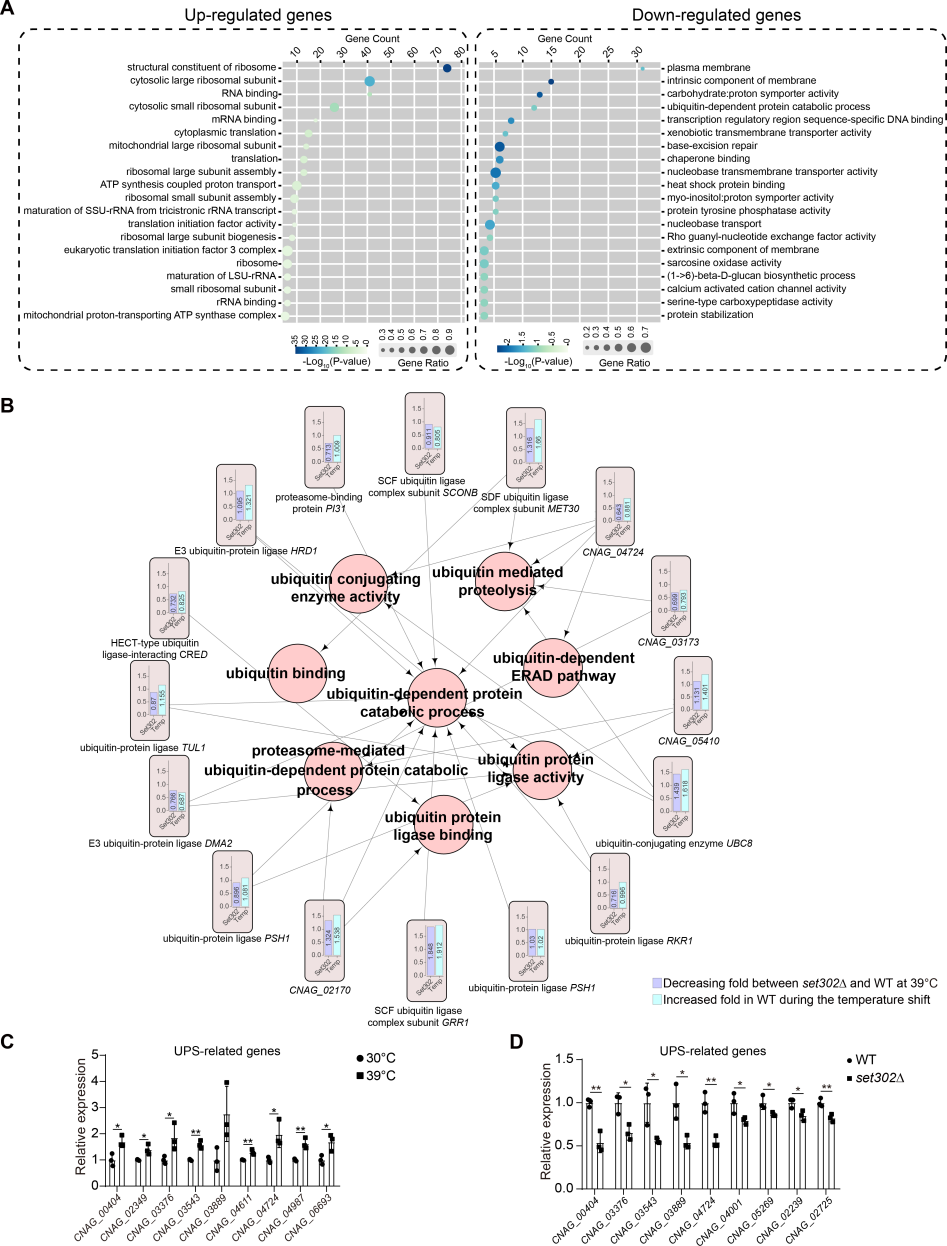
The expression of UPS components is influenced by Set302 deletion at high temperatures. (**A**) GO analysis of genes responsive to heat stress and with altered expression levels in the deletion of Set302 at 39°C but not at 30°C. GO was performed using KOBAS 3.0. (**B**) Schematic representation of gene expression and the relationship of GO terms. In the orange box, the left chart represents the reduced fold change of gene expression in the *set302∆* strain relative to the wild-type strain at 39°C, and the right chart represents the increased fold change of gene expression in the wild-type strain in response to heat stress. (**C**) Quantitative RT-PCR was performed using total RNA from wild-type strains at 30°C or 39°C, respectively, and the gene expression of representative UPS genes was measured. (**D**) Quantitative RT-PCR was performed using total RNA from wild-type and *set302∆* strains at 39°C and the gene expression of representative UPS genes was measured.

To clarify how Set302 affects thermotolerance in *C. neoformans*, we examined the 627 up-regulated and 1,059 down-regulated genes in *set302∆* strain at 39°C, but not at 30°C. A Gene Ontology (GO) analysis revealed that Set302 has a broad effect on various primary biological functions and cellular components, including translation, RNA binding, plasma membrane, and ribosome at 39°C (Fig. 4A; Table S2). However, no difference in membrane integrity was detected between wild-type and *set302∆* strains (Fig. S2A). Notably, a subset of genes positively regulated by Set302 were enriched in the ubiquitin-dependent protein catabolic process, which is related to UPS (Fig. 4A; Table S2). UPS has been reported to play an important role in fungal survival under various conditions (47–49). Further investigation showed that the expression of UPS-related genes was positively regulated by Set302 at 39°C. Moreover, these genes were associated with terms, such as ubiquitin-mediated proteolysis and ubiquitin-conjugating enzyme activity, highlighting their functional connectivity (Fig. 4B). Surprisingly, we observed a similar change in their expression levels under heat stress to that observed in *set302∆* strain at 39°C. (Fig. 4B). This observation was further confirmed by quantitative reverse transcription polymerase chain reaction (qRT-PCR), which demonstrated that the expression of these genes increased in response to heat stress (Fig. 4C) and decreased in *set302∆* strain at 39°C (Fig. 4D). These findings suggest a potential relationship between the function of UPS, Set302, and thermotolerance.

### The role of Set302 in the degradation of misfolded proteins under heat stress

**Fig 5:**
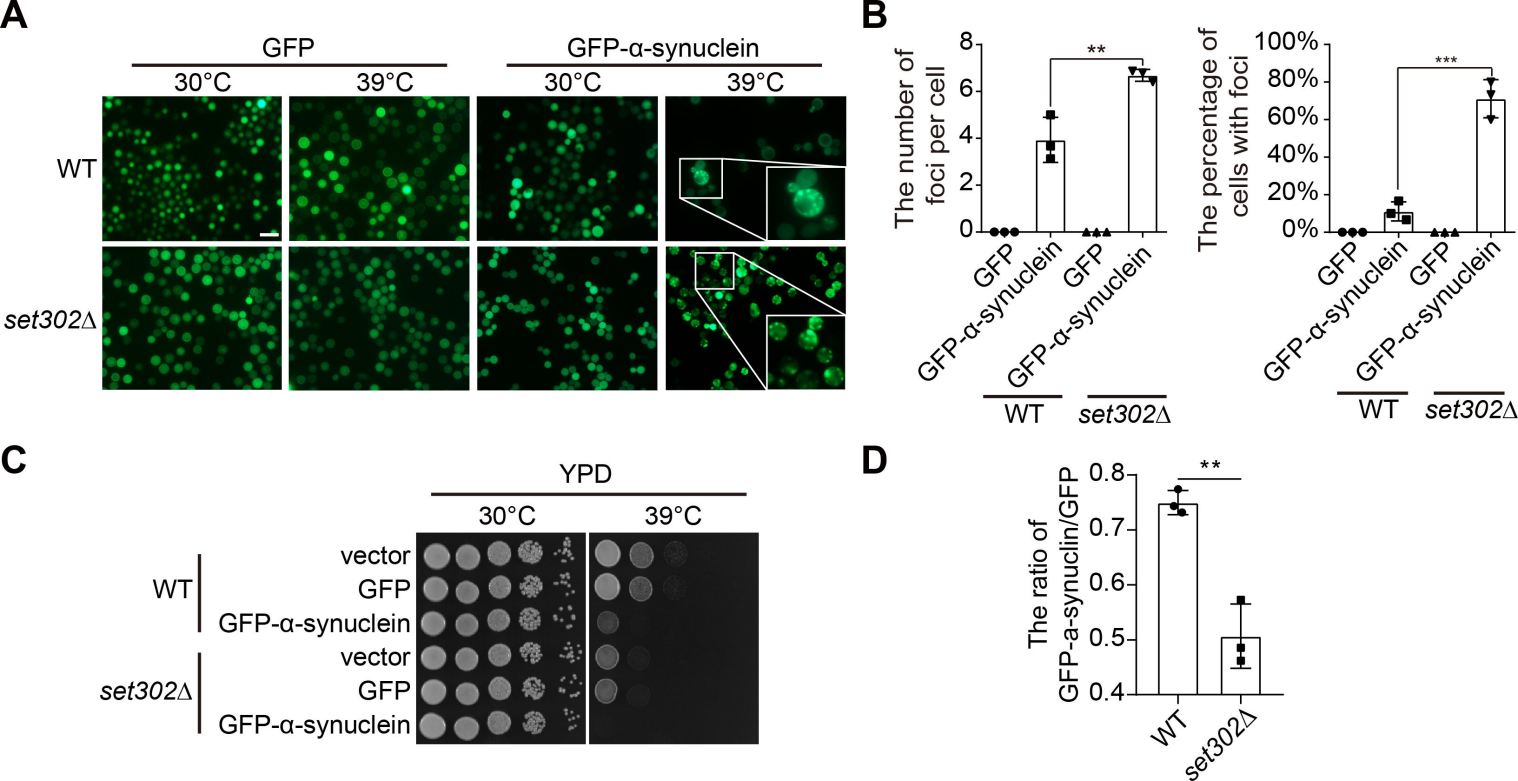
The lack of Set302 results in the declined degradation process of misfolded protein under heat stress. (**A**) The representative fluorescence photographs of GFP or GFP-labeled α-synuclein overexpressed in wild-type and *set302∆* strains at 30°C and 39°C. Fungal cells were harvested at 30°C or 39°C and observed by fluorescence microscopy. Scale bar = 10 microns. (**B**) The statistics of fluorescence foci number per cell and the percentage of cells with fluorescence foci from wild-type and *set302∆* strains overexpressing GFP or GFP-labeled α-synuclein at 39°C, respectively. (**C**) Spot dilution assays with wild-type and *set302∆* strains overexpressing the control vector, GFP or GFP-labeled α-synuclein were performed onto YPD agar and incubated at 30°C or 39°C for 2 days, respectively. (**D**) The relative quantitative analysis of wild-type and *set302∆* strains with overexpressed GFP or GFP-labeled α-synuclein colony spot at 39°C. The signal intensity of colony spot was calculated using ImageJ and the intensity ratio of wild-type or *set302∆* strains with GFP-labeled α-synuclein relative to those with GFP was calculated.

UPS has been implicated in regulating the degradation of misfolded proteins. For example, α-synuclein is a protein associated with Parkinson’s disease that undergoes misfolding and is primarily eliminated by UPS (50). Additionally, the yeast model overexpressing α-synuclein has been used to investigate the accumulation of misfolded proteins and the resulting cellular toxicity (51). To investigate whether the degradation of misfolded proteins regulated by Set302-mediated UPS function contributes to thermotolerance in *C. neoformans*, we created a GFP-labeled α-synuclein-overexpressed model in wild-type and *set302∆* strains. In this *C. neoformans* model, the fluorescence foci of the aggregates were observed if GFP-labeled α-synuclein was misfolded. Almost no fluorescent aggregates were caused by misfolded α-synuclein at 30°C in both strains, suggesting that misfolded α-synuclein rarely forms under normal conditions. However, at 39°C, a small fraction of wild-type strain cells contained fluorescent aggregates, suggesting that this elevated temperature leads to misfolding of α-synuclein (Fig. 5A). Wild-type strain exhibited approximately 10% of cells with foci, and of those with foci, the average number of foci per cell was about 4. In contrast, the percentage of cells with foci for *set302∆* strain was about 70%, and the average number of foci per cell in those cells with foci was about 6, which were both higher than those of wild-type, suggesting that the lack of *SET302* contributed to the increased aggregate formation (Fig. 5B). To assess the effect of misfolded protein formation on the ability of the fungus to grow, we examined the phenotypes of wild-type and *set302∆* strain overexpressing α-synuclein at 30°C and 39°C. As a result, no difference in growing ability was observed between wild-type and *set302∆* strain at 30°C, which was consistent with the observation of fluorescent aggregates. However, the misfolded protein aggregation strikingly reduced the growing ability of *C. neoformans* at 39°C (Fig. 5C). Notably, inhibited growth was more pronounced in the *set302∆* strain than in the wild-type strain under heat stress (Fig. 5C and D). Collectively, these results indicate that the absence of *SET302* results in a higher accumulation of misfolded proteins, such as α-synuclein, under heat stress.

### The deletion of Set302 leads to decreased growth of *C. neoformans* under proteasome stress

**Fig 6:**
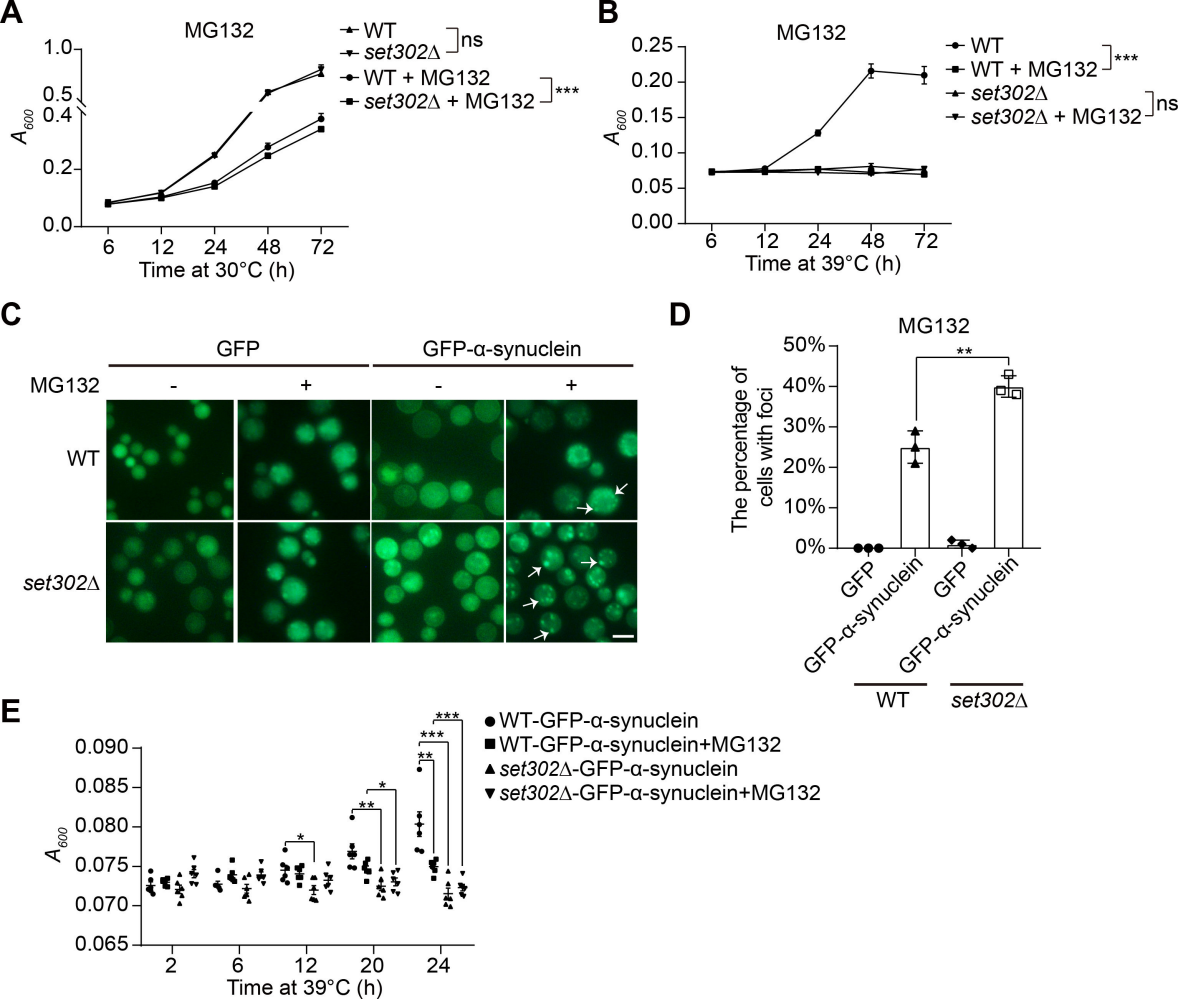
The deletion of Set302 leads to decreased growth for *C. neoformans* under proteasome stress. (**A**) Growth curve of fungal cells in liquid cultures. Wild-type and *set302∆* strains were grown in YPD liquid media with or without 100 µM MG132 at 30°C. Cell growths (absorbance at 600 nm) were quantified at indicated time points. (**B**) Growth curve of fungal cells in liquid cultures. Wild-type and *set302∆* strains were grown in YPD liquid media with or without 100 µM MG132 at 39°C. Cell growths (absorbance at 600 nm) were measured at indicated time points. (**C**) The representative fluorescence photographs of GFP or GFP-labeled α-synuclein overexpressed in wild-type and *set302∆* strains with or without 100 µM MG132. Fungal cells were harvested and observed by fluorescence microscopy. Scale bar = 5 microns. (**D**) The statistics of the percentage of cells with fluorescence foci from wild-type and *set302∆* strains overexpressing GFP or GFP-labeled α-synuclein with 100 µM MG132 treatment, respectively. (**E**) Quantification of fungal growth in liquid cultures. Wild-type and *set302∆* strains overexpressing GFP-labeled α-synuclein were grown in YPD liquid media with or without 100 µM MG132 at 39°C. Cell growths (absorbance at 600 nm) were measured at indicated time points.

Heat stress influences UPS function by affecting its activity and efficiency (52, 53). Our data suggest that Set302 is important to the UPS pathway by affecting the degradation of misfolded proteins under heat stress in *C. neoformans*; thus, we speculated that it also functions under other UPS stress. To explore this possibility, we assessed growth in the presence of the proteasome inhibitor MG132, which is commonly used to disturb ubiquitin-mediated protein degradation (54). The results showed that the *set302∆* strain had slightly lower growth than the wild-type strain when treated with MG132 (Fig. 6A). Additionally, MG132 effectively inhibited the growth of the wild-type strain under heat stress (Fig. 6B), indicating that UPS plays a role in the thermotolerance of *C. neoformans*. The fluorescence observations of GFP-labeled α-synuclein revealed that MG132 exacerbated the formation of aggregates similar to heat stress, indicating that the elimination of misfolded proteins, such as α-synuclein, is also dependent on UPS function in *C. neoformans* (Fig. 6C). The absence of *SET302* caused a higher percentage of cells with fluorescent aggregates, exhibiting a similar effect as heat stress (Fig. 6D). MG132 also inhibited the growth of wild-type strain overexpressing α-synuclein significantly more than *set302∆* strain overexpressing α-synuclein at 39°C (Fig. 6E). We hypothesized that this was attributed to the finding that the functional integrity of UPS in wild-type strain is stronger than that of *set302∆* strain, which was damaged under heat stress, and therefore, *set302∆* strain was less inhibited than wild-type when treated with MG132. Wild-type strain overexpressing GFP-labeled α-synuclein also displayed stronger growth than the *set302∆* strain without MG132 treatment at 39°C (Fig. 6E). This observation agrees with the findings from the spot-assay of these two strains at 39°C. Taken together, these results suggest that Set302 contributes to the degradation of misfolded proteins when cells are exposed to various UPS stress conditions.

### The lack of Set302 reduces virulence and tissue colonization of *C. neoformans*

**Fig 7:**
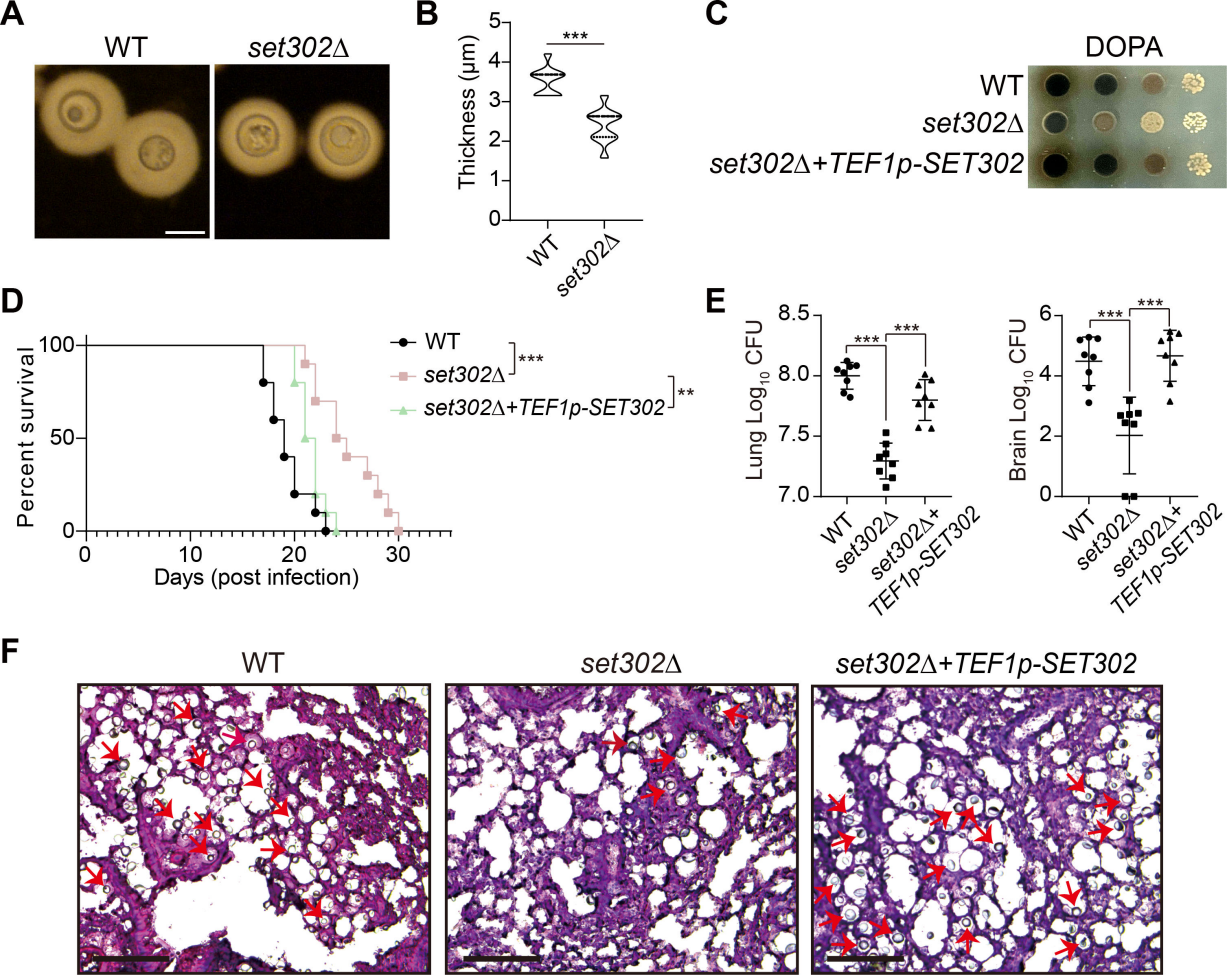
The absence of Set302 reduced the formation of virulence factors and the pathogenicity of *C. neoformans*. (**A**) Representative microscopy images of capsule assay. Strains were grown in Dulbecco’s Modified Eagle Medium (DMEM)+10% fetal bovine serum (FBS) medium at 37°C for 2 days followed by India ink staining and photographing. Scale bars = 5 microns. (**B**) The capsule sizes of wild-type and *set302∆* strains (*n* = 20) were measured and compared. (**C**) The melanin production assay of wild-type, *set302∆,* and *set302∆+TEF1p-SET302* strains. Strains were spotted on the YPD agar plate with L-DOPA and incubated at 37°C for 2 days. (**D**) Animal survival analysis and the Kaplan-Meier survival plot of wild-type, *set302∆,* and *set302∆+TEF1p-SET302* strains. Cells were grown overnight in YPD liquid medium, harvested, washed with PBS, and inoculated into mice via intranasal instillation. Animals were sacrificed at defined endpoints of mortality and survival was plotted. Significance was determined using the Log-rank (Mantel-Cox) test. (**E**) The colony forms units of surviving fungal cells per gram from infected organs. The infected lung and brain with wild-type, *set302∆,* and *set302∆+TEF1p-SET302* strains were extracted, homogenized, and plated onto YPD agar. The colony-forming units were counted and normalized to organ weight. (**F**) Histopathological staining of lung tissues infected by wild-type, *set302∆,* and *set302∆+TEF1p-SET302* strains with Periodic Acid-Schiff. The lung tissues were captured under a ×10 lens (scale bars = 100 µm). Fungal cells were indicated with arrows.

In addition to thermotolerance, capsule, and melanin are also crucial to the pathogenicity of *C. neoformans* (55). The results of the microscopic images and quantitative analysis of capsule size showed that the *set302∆* strain displayed a smaller surface capsule than the wild-type, suggesting that Set302 contributes to capsule size (Fig. 7A and B). Defective melanin production was also observed in the *set302∆* strain compared to the wild-type and the *SET302* complement rescued this melanin defect in the *set302∆* strain (Fig. 7C). By analyzing the differentially expressed genes in RNA-seq and comparing them with the capsule- and melanin-related genes gained from Cryptonet database, we found that the lack of *SET302* changed the expression of six capsule formation-related genes and six melanin production-related genes only at high temperature, but not at normal condition. Among these genes, five capsule formation-related genes and five melanin production-related genes are responsive to heat stress (Fig. S4). This result suggests that Set302 may affect these virulence factors by influencing the expression level of related genes under heat stress such as in the host environment, thereby contributing to the pathogenicity of *C. neoformans*.

Due to the defective phenotype of these critical virulence factors of the *set302∆* strain, we aimed to investigate the effect of Set302 on *C. neoformans* pathogenicity. Using a murine inhalation model of cryptococcosis, we found that female C57BL/6 mice with an intranasal infection by *set302∆* strain showed significantly prolonged survival than those by wild-type, and rescuing the *SET302* gene overcame the reduced virulence caused by the lack of *SET302* (Fig. 7D). Furthermore, *set302∆* strain exhibited markedly reduced colony forming units in the lung and brain of infected mice compared to wild-type strain, and the rescue of *SET302* increased the colonization capacity of *set302∆* strain (Fig. 7E). The histopathological assessment with Periodic Acid-Schiff staining showed fewer fungal cells in the lung of mice with the infection by *set302∆* strain (Fig. 7F). Overall, these results reveal that Set302 contributes to the colonization in the infected tissues and the pathogenicity of *C. neoformans* to the host.

## DISCUSSION

In this study, we aimed to characterize the function of the histone deacetylase complex Set3C in response to heat stress in *C. neoformans*. In *S. cerevisiae*, the core subunit Set3 is vital to the assembly and integrity of the Set3C complex (31). The Set3 protein contains a SET domain, which potentially mediates protein-protein interaction, and a PHD domain, which binds the methylated tails of histone 3 (32, 56). Analysis of the Set3 protein homolog Set302 in *C. neoformans* revealed the presence of both the PHD and SET domains. Interestingly, the PHD and SET domains are commonly present, except in *R. delemar*, where the PHD domain is absent, suggesting that *R. delemar* Set3 may have a distinct role from other fungi owing to the lack of a PHD domain. One study showed that the phylogenies based on SET domains in Ascomycetes do not appear to consistently support any specific evolutionary relationship among fungi, animals, and plants. An evolutionary tree analysis of the Set3 proteins from Saccharomycotina fungi indicated a high degree of homology between Set3 and Set4, which could be due to the whole-genome duplication event in the history of Saccharomyces (57). Our study analyzed the phylogenetic tree of the Set3 proteins from different fungal phyla, including Saccharomycotina, and showed that the Set3 proteins tended to cluster based on evolutionary branches, suggesting a higher degree of evolutionary conservation among the Set3 proteins. It is noteworthy that the Set3 proteins from Basidiomycota, including *C. neoformans*, *Ustilago maydis*, and *Coprinopsis cinerea*, have longer protein sequences than those in other fungi, suggesting subtle differences in the Set3 proteins between Basidiomycota and other phyla.

Previous findings demonstrated that in *S. cerevisiae*, the deletion of Set3 does not affect growth or stress response (31). However, this leads to faster meiosis I, meiosis II, and ascus formation progression. In *Magnaporthe oryzae*, the absence of *SET3* strongly reduces virulence and impairs conidiation (41). In *Fusarium graminearum*, Set3C affects virulence through the interaction with the cAMP-PKA pathway (58). In the human pathogenic fungus *C. albicans*, the Set3C complex plays a crucial role in regulating switching modulation and yeast-filament transition caused by a hyperactive cAMP/PKA pathway, thereby affecting the virulence of *C. albicans* (43, 44). However, based on the transcriptomics data, we found that the deletion of *SET302* in *C. neoformans* had little effect on the cAMP/PKA pathway. Some minor changes in gene expression, such as GPR4 and Gpa1 occurred, but most cAMP/PKA pathway-related genes, particularly the core regulator of cAMP production, Cac1, and the downstream execution factor, Pka, remained unchanged (Table S1).

In this study, we found that the *set302∆* strain was equally viable with the wild-type, exhibiting a similar phenotype to those in *S. cerevisiae*, *M. oryzae*, and *C. albicans*. However, the *set302∆* strain displayed defective thermotolerance in solid and liquid media conditions and decreased viability under the extreme temperature pretreatment, indicating that Set3C-mediated thermotolerance is controlled by the ability to survive, not the growth rate. We also determined that the deletion of *SET302* reduced but did not abolish the thermotolerance of *C. neoformans*, suggesting that Set3C complex is not indispensable for thermotolerance, but plays a partial role and is redundant with other factors. Intensive efforts have been made to elucidate the key regulators governing thermotolerance in *C. neoformans* (24, 25, 27, 29). However, Set302 plays a role in thermotolerance independent of these pathways mentioned above.

UPS is critical for selectively digesting misfolded or useless proteins marked by ubiquitin in all eukaryotes to maintain a properly functioning proteome. UPS involves the attachment of ubiquitin to target proteins, which are then recognized and degraded by the 26S proteasome (59–62). In pathogenic fungi, UPS regulates the expression and activity of many pathogenic factors that are crucial for virulence, stress response, and fungal morphology (48, 62, 63). In this study, we found that multiple UPS-related pathways responded to heat stress and were regulated by Set302 at high temperatures. Heat stress can lead to abnormal protein folding, resulting in an imbalance of protein homeostasis (46) and UPS-mediated degradation is the predominant mechanism for the clearance of misfolded proteins, such as α-synuclein (50, 64, 65). In this study, we found that the lack of *SET302* led to increased formation of α-synuclein aggregates under heat stress. Research in the model organism *S. cerevisiae* has demonstrated that the formation of misfolded proteins impairs growing ability (66). Similarly, protein aggregation caused by misfolded proteins significantly inhibits the thermotolerance of *C. neoformans*, and the *set302∆* strain displayed a greater sensitivity to protein aggregation-induced damage than the wild-type strain, suggesting a role of Set302 in protecting *C. neoformans* from damage caused by misfolded proteins under heat stress. We speculated that Set302 is a potential gene regulator that maintains UPS function under heat stress by affecting the expression of UPS-related genes, including E3 ubiquitin-protein ligases and proteasome subunits.

Heat stress induces the ubiquitination of many cellular proteins followed by proteasomal degradation (67). Previous studies have reported that the α2 subunit of the 26S proteasome and various E3 ubiquitin ligases are associated with enhanced thermotolerance (68–70). Additionally, heat stress can function as a UPS stressor by inactivating the proteasome and inhibiting the assembly of the proteasome complex (71). We demonstrated here that the inhibition of UPS with MG132 decreased *C. neoformans* thermotolerance. Notably, the *set302∆* strain exhibited increased protein aggregation formed by α-synuclein compared to the wild-type when treated with MG132, and this protein aggregation exerted a stronger inhibitory effect on thermotolerance of the *set302∆* strain, similar to the phenotype observed under heat stress conditions, suggesting that Set302 is a universal regulator for UPS function in the elimination of misfolded proteins under different UPS stressors. However, explaining how Set302 affects UPS function will require further studies.

Many studies have shown a close relationship between the major virulence factors, including thermotolerance, capsule, and melanin, and the pathogenicity of *C. neoformans* (17, 18, 20, 21, 29, 55). In this study, the *set302∆* strain also exhibited a defective capsule and melanin formation. Oliver W. Liu et al. showed that Set302 has a slight inhibitory effect on capsule formation (72), whereas Yunfang Meng et al. reported that *set302∆* strain has no apparent capsular defect (45). We speculated that this difference is due to differences in the capsule-inducing conditions. The capsule-inducing conditions used in our study that mimic the host environment led to differences in capsule formation. Furthermore, in a murine inhalation model, the *set302∆* strain with deficiencies in these virulence factors displayed significantly reduced virulence and a lower fungal burden in lung and brain tissues compared to the wild-type strain. These findings are consistent with the observed effects on the pathogenicity of *M. oryzae* and *C. albicans*, further illustrating the significance of Set3C complex in the pathogenicity of pathogenic fungi. Although the deletion of *SET302* led to defective thermotolerance, Set302 was not particularly critical for thermotolerance, suggesting that Set302 may play a partial role in thermotolerance and that the reduction in pathogenicity may result from a combination of these major virulence factors, including thermotolerance, capsule, and melanin.

## MATERIALS AND METHODS

### Fungal strains and culture conditions

*C. neoformans* var.grubii wild-type strain H99 was used for analysis in this study. Strains were cultured in YPD (1% yeast extract, 2% peptone, 2% dextrose) medium. For the selection of transformants, G418 and hygromycin B were added to the YPD agar medium with a final concentration of 200 µg/mL and 200 units/mL, respectively. The strains were grown at 30°C for normal culture unless it was stated at a specific temperature. The stress medium was supplemented with 0.4 M calcium chloride, 0.02% SDS, 20 mM DTT, or 100 µg/mL cyclosporin A. 100 μM MG132 was added to the YPD medium for proteasome activity detection. L-DOPA agar and Dulbecco’s Modified Eagle Medium (DMEM) supplemented with 10% of fetal bovine serum (FBS) were used for melanin and capsule production. For the growth curve experiment, the strains were cultured in a YPD medium with the initial OD_600_ value of 0.05 and incubated at indicated conditions. The OD_600_ value was measured using a MultiskanGO microplate reader (Thermo).

### Construction of fungal strains

The strains are listed in Table S3. The manipulation of *C. neoformans* and protocols were described previously (73). *SET302* gene (*CNAG_06591*) was knocked out by the homologous replacement of its open reading frame with a drug-resistance cassette. Primer pairs CDp2419/CDp2420 and CDp2421/CDp2422 amplified SET302’s flanking regions, while M13F/M13R primers amplified the NEO marker from the pHA-NEO vector. Then *set302∆::NEO* construct was obtained by CDp2419/CDp2422 primer pair and transformed into H99 strain. CDp2477/CDp2478 primer pair was used for the diagnostic PCR.

For *SET302* complementation, *SET302* upstream and *TEF1p* sequences were amplified using GXD85/86 and GXD87/88 primer pairs and cloned into the pHA-HYG vector. The open reading frame of *SET302* and *SET302* downstream amplified using GXD91/92 primer pair and GXD89/90 primer pair were cloned into the above-constructed vector. The cassette was amplified using the GXD85/90 primer pair and transformed into the *set302∆* strain. Diagnostic PCR was performed using the GXD87/CDp2478 primer pair.

For the GFP overexpressing strain, the GFP coding sequence was amplified using the CDp2665/CDp2671 primer pair and cloned at the *Pac*I site of the *pHYG* plasmid. For GFP-α-synuclein overexpressing strain, GFP and -α-synuclein coding sequences were amplified using CDp2665/CDp2666 and CDp2674/CDp2673 primer pair and then cloned at the *Pac*I site of the *pHYG* plasmid. The reconstructed plasmids were transformed into H99 or *set302∆* strains. All primers were listed in Table S4.

### *In vitro* assay for thermotolerance at extreme temperature

The strains were cultured overnight at 30°C. Then the strains were inoculated to OD_600_ = 1 in the preheated tubes at a 50°C hot block. Aliquots were removed at the indicated time and placed immediately on ice. A hundred dilutions were plotted onto YPD plates and incubated at 30°C for 2 days to determine the number of colony-forming units.

### The fluorescence signal detection

GFP or GFP-α-synuclein overexpressing strains were cultured in a YPD medium at indicated conditions. Fungal cultures were washed twice, resuspended by sterile PBS, and imaged using a Nikon DS-Ri1 microscopy. The percentage of fungal cells with fluorescence foci was quantified from 30 cells with GFP fluorescence signal, and the number of fluorescence foci per cell was counted from 15 cells with foci, both from three independent experiments.

### Total RNA preparation and quantitative RT-PCR

The strains were grown to the mid-log phase in YPD medium at 30°C or 39°C. Cells were centrifuged at 1,000 *× g* for 5 minutes at 4°C and washed twice with ice-cold ddH2O. Total RNA was extracted using the Total RNA Kit I (Omega). cDNA was synthesized using the Reverse Transcript All-in-one Mix (Mona), followed by genomic DNA removal using TURBO DNA-free (Invitrogen). The primers are listed in Table S4. The CFX96 real-time system (Bio-Rad) was used for data acquisition. ACT1 was used as a normalization control. The relative gene expression levels were calculated using the ΔΔCt method.

### Phylogenetic and protein domain analysis

The domain analysis was performed using Interpro (http://www.ebi.ac.uk/interpro/). The phylogenetic tree was constructed by MEGA5 using the neighbor-joining method and displayed using Figtree software. Multiple sequence alignment was performed using Clustal Omega (https://www.ebi.ac.uk/Tools/msa/clustalo/). The conservation sites were calculated using Jalview software. The Blastp hits were calculated by NCBI using the protein sequence of the SET domain of *S.cerevisiae* Set3 (YKR029C) as the template.

### RNA-seq and data analysis

Three independent samples of wild-type and *set302∆* strains were inoculated in 10 mL YPD broth at 30°C overnight. The fungal strains were subcultured in 100 mL YPD medium with an OD_600_ of 0.2 until the OD_600_ reached 1 at 30°C or 39°C, respectively. The fungal strains were washed with PBS three times and frozen in liquid nitrogen. Total RNA was extracted using TRIzol regent (Invitrogen Life Technologies). The process protocol for RNA-seq and data analysis was described previously (29).

### Animal experiments

Animal survival assay, fungal burden analysis, and histopathology analysis were conducted as described previously (29).

### Bioinformatic methods

GO analysis was conducted using KOBAS 3.0. Heatmap was generated using the pheatmap package and the Fragments Per Kilobase of transcript per Million mapped reads (FPKM) values was transformed using the Log_2_(FPKM + 1) formula. The correlation analysis was calculated using the corrplot package. Packages were operated in R.3.4.3. The Upset plot was conducted using Evenn.

### Statistical analysis

Statistical analysis was conducted with GraphPad Prism 8.0.1. Two-group comparisons and mouse survival data were analyzed by two-tailed Student’s *t*-test and Log-rank test, respectively. Growth curve differences were assessed using two-way ANOVA. A *P*-value <0.05 indicated significance (*P < 0.05; **P < 0.01; ***P < 0.001). In transcriptome analysis, genes with fold changes >1.5 or <0.67 and *P* < 0.05 were considered differentially expressed.

## Data Availability

The transcriptome data is deposited in NCBI’s Gene Expression Omnibus (GEO) and can be assessed through GEO series accession ID GEO GSE242109.

## SUPPLEMENTAL MATERIAL

Supplemental figures spectrum.04202-23-s0001.docx Fig. S1-S4.

Table S1 spectrum.04202-23-s0002.xlsx Transcriptome analysis in *C. neoformans knock-outs or wild-type cells at indicated temperature.*

Table S2 spectrum.04202-23-s0003.xlsx GO analysis of DEG in *C. neoformans* knock-outs or wild-type cells.

Table S3 spectrum.04202-23-s0004.xlsx Strains used in this study.

Table S4 spectrum.04202-23-s0005.xlsx Primers used in this study.

ASM does not own the copyrights to Supplemental Material that may be linked to, or accessed through, an article. The authors have granted ASM a non-exclusive, world-wide license to publish the Supplemental Material files. Please contact the corresponding author directly for reuse.

## References

[1] Garcia-Bustos V, Cabañero-Navalon MD, Ruiz-Gaitán A, Salavert M, Tormo-Mas MÁ, Pemán J. 2023. Climate change, animals, and Candida auris: insights into the ecological niche of a new species from a one health approach. Clin Microbiol Infect 29:858–862. doi:10.1016/j.cmi.2023.03.016 36934871

[2] Suo C, Gao Y, Ding C, Sun T. 2023. The function and regulation of heat shock transcription factor in Cryptococcus. Front Cell Infect Microbiol 13:1195968. doi:10.3389/fcimb.2023.1195968 37168390 PMC10165103

[3] Xiao W, Zhang J, Huang J, Xin C, Li MJ, Song Z. 2022. Response and regulatory mechanisms of heat resistance in pathogenic fungi. Appl Microbiol Biotechnol 106:5415–5431. doi:10.1007/s00253-022-12119-2 35941254 PMC9360699

[4] Allsup CM, George I, Lankau RA. 2023. Shifting microbial communities can enhance tree tolerance to changing climates. Science 380:835–840. doi:10.1126/science.adf2027 37228219

[5] de S Araújo GR, Souza W de, Frases S. 2017. The hidden pathogenic potential of environmental fungi. Future Microbiol 12:1533–1540. doi:10.2217/fmb-2017-0124 29168657

[6] Bergman A, Casadevall A. 2010. Mammalian endothermy optimally restricts fungi and metabolic costs. mBio 1:e00212-10. doi:10.1128/mBio.00212-10 21060737 PMC2975364

[7] Robert VA, Casadevall A. 2009. Vertebrate endothermy restricts most fungi as potential pathogens. J Infect Dis 200:1623–1626. doi:10.1086/644642 19827944

[8] Qi W, Acosta-Zaldivar M, Flanagan PR, Liu N-N, Jani N, Fierro JF, Andrés MT, Moran GP, Köhler JR. 2022. Stress- and metabolic responses of Candida albicans require Tor1 kinase N-terminal HEAT repeats. PLoS Pathog 18:e1010089. doi:10.1371/journal.ppat.1010089 35687592 PMC9223334

[9] Gostinčar C, Zajc J, Lenassi M, Plemenitaš A, de Hoog S, Al-Hatmi AMS, Gunde-Cimerman N. 2018. Fungi between extremotolerance and opportunistic pathogenicity on humans. Fungal Diversity 93:195–213. doi:10.1007/s13225-018-0414-8

[10] Egidi E, Delgado-Baquerizo M, Plett JM, Wang J, Eldridge DJ, Bardgett RD, Maestre FT, Singh BK. 2019. A few ascomycota taxa dominate soil fungal communities worldwide. Nat Commun 10:2369. doi:10.1038/s41467-019-10373-z 31147554 PMC6542806

[11] Robert V, Cardinali G, Casadevall A. 2015. Distribution and impact of yeast thermal tolerance permissive for mammalian infection. BMC Biol 13:18. doi:10.1186/s12915-015-0127-3 25762372 PMC4381509

[12] Spivak ES, Hanson KE. 2018. Candida auris: an emerging fungal pathogen. J Clin Microbiol 56:e01588-17. doi:10.1128/JCM.01588-17 29167291 PMC5786713

[13] Casadevall A, Kontoyiannis DP, Robert V. 2019. On the emergence of Candida auris: climate change, azoles, swamps, and birds. mBio 10:e01397-19. doi:10.1128/mBio.01397-19 31337723 PMC6650554

[14] Zhao Y, Ye L, Zhao F, Zhang L, Lu Z, Chu T, Wang S, Liu Z, Sun Y, Chen M, Liao G, Ding C, Xu Y, Liao W, Wang L. 2023. Cryptococcus neoformans, a global threat to human health. Infect Dis Poverty 12:20. doi:10.1186/s40249-023-01073-4 36932414 PMC10020775

[15] Rajasingham R, Smith RM, Park BJ, Jarvis JN, Govender NP, Chiller TM, Denning DW, Loyse A, Boulware DR. 2017. Global burden of disease of HIV-associated cryptococcal meningitis: an updated analysis. Lancet Infect Dis 17:873–881. doi:10.1016/S1473-3099(17)30243-8 28483415 PMC5818156

[16] May RC, Stone NRH, Wiesner DL, Bicanic T, Nielsen K. 2016. Cryptococcus: from environmental saprophyte to global pathogen. Nat Rev Microbiol 14:106–117. doi:10.1038/nrmicro.2015.6 26685750 PMC5019959

[17] Perfect JR. 2006. Cryptococcus neoformans: the yeast that likes it hot. FEMS Yeast Res 6:463–468. doi:10.1111/j.1567-1364.2006.00051.x 16696642

[18] Steen BR, Lian T, Zuyderduyn S, MacDonald WK, Marra M, Jones SJM, Kronstad JW. 2002. Temperature-regulated transcription in the pathogenic fungus Cryptococcus neoformans. Genome Res 12:1386–1400. doi:10.1101/gr.80202 12213776 PMC186651

[19] Montoya MC, Magwene PM, Perfect JR. 2021. Associations between Cryptococcus genotypes, phenotypes, and clinical parameters of human disease: a review. J Fungi (Basel) 7:260. doi:10.3390/jof7040260 33808500 PMC8067209

[20] Garcia-Solache MA, Izquierdo-Garcia D, Smith C, Bergman A, Casadevall A. 2013. Fungal virulence in a lepidopteran model is an emergent property with deterministic features. mBio 4:e00100-13. doi:10.1128/mBio.00100-13 23631914 PMC3648900

[21] Bloom ALM, Jin RM, Leipheimer J, Bard JE, Yergeau D, Wohlfert EA, Panepinto JC. 2019. Thermotolerance in the pathogen Cryptococcus neoformans is linked to antigen masking via mRNA decay-dependent reprogramming. Nat Commun 10:4950. doi:10.1038/s41467-019-12907-x 31666517 PMC6821889

[22] Dai K, Feng Z, Hu T, Su Z, Yuan D, Qin BE, Gu M, Peng F, Jiang Y. 2023. Seasonality and meteorological factors of HIV-negative cryptococcal meningitis in Guangdong province, China. Mycoses 66:1003–1011. doi:10.1111/myc.13647 37563970

[23] Hu G, Horianopoulos L, Sánchez-León E, Caza M, Jung W, Kronstad JW. 2021. The monothiol glutaredoxin Grx4 influences thermotolerance, cell wall integrity, and Mpk1 signaling in Cryptococcus neoformans. G3 (Bethesda) 11:jkab322. doi:10.1093/g3journal/jkab322 34542604 PMC8527476

[24] Yang DH, Jung KW, Bang S, Lee JW, Song MH, Floyd-Averette A, Festa RA, Ianiri G, Idnurm A, Thiele DJ, Heitman J, Bahn YS. 2017. Rewiring of signaling networks modulating thermotolerance in the human pathogen Cryptococcus neoformans. Genetics 205:201–219. doi:10.1534/genetics.116.190595 27866167 PMC5223503

[25] Park H-S, Chow EWL, Fu C, Soderblom EJ, Moseley MA, Heitman J, Cardenas ME. 2016. Calcineurin targets involved in stress survival and fungal virulence. PLoS Pathog 12:e1005873. doi:10.1371/journal.ppat.1005873 27611567 PMC5017699

[26] Leach MD, Cowen LE. 2014. To sense or die: mechanisms of temperature sensing in fungal pathogens. Curr Fungal Infect Rep 8:185–191. doi:10.1007/s12281-014-0182-1

[27] Cheon SA, Jung KW, Chen YL, Heitman J, Bahn YS, Kang HA. 2011. Unique evolution of the UPR pathway with a novel bZIP transcription factor, Hxl1, for controlling pathogenicity of Cryptococcus neoformans. PLoS Pathog 7:e1002177. doi:10.1371/journal.ppat.1002177 21852949 PMC3154848

[28] Probst C, Hallas-Møller M, Ipsen JØ, Brooks JT, Andersen K, Haon M, Berrin J-G, Martens HJ, Nichols CB, Johansen KS, Alspaugh JA. 2023. A fungal lytic polysaccharide monooxygenase is required for cell wall integrity, thermotolerance, and virulence of the fungal human pathogen Cryptococcus neoformans. PLoS Pathog 19:e1010946. doi:10.1371/journal.ppat.1010946 37099613 PMC10166503

[29] Gao X, Fu Y, Sun S, Gu T, Li Y, Sun T, Li H, Du W, Suo C, Li C, Gao Y, Meng Y, Ni Y, Yang S, Lan T, Sai S, Li J, Yu K, Wang P, Ding C. 2022. Cryptococcal Hsf3 controls intramitochondrial ROS homeostasis by regulating the respiratory process. Nat Commun 13:5407. doi:10.1038/s41467-022-33168-1 36109512 PMC9477856

[30] Reyes AA, Fishbain S, He Y. 2022. Structural and functional analysis of the SET3 histone deacetylase complex. Acta Crystallogr F Struct Biol Commun 78:113–118. doi:10.1107/S2053230X22000553 35234136 PMC8900736

[31] Pijnappel WW, Schaft D, Roguev A, Shevchenko A, Tekotte H, Wilm M, Rigaut G, Séraphin B, Aasland R, Stewart AF. 2001. The S. cerevisiae SET3 complex includes two histone deacetylases, Hos2 and Hst1, and is a meiotic-specific repressor of the sporulation gene program. Genes Dev 15:2991–3004. doi:10.1101/gad.207401 11711434 PMC312828

[32] Kim T, Buratowski S. 2009. Dimethylation of H3K4 by Set1 recruits the Set3 histone deacetylase complex to 5' transcribed regions. Cell 137:259–272. doi:10.1016/j.cell.2009.02.045 19379692 PMC2802783

[33] Jones RS, Gelbart WM. 1993. The Drosophila polycomb-group gene enhancer of zeste contains a region with sequence similarity to trithorax. Mol Cell Biol 13:6357–6366. doi:10.1128/mcb.13.10.6357-6366.1993 8413234 PMC364694

[34] Tschiersch B, Hofmann A, Krauss V, Dorn R, Korge G, Reuter G. 1994. The protein encoded by the Drosophila position-effect variegation suppressor gene Su(var)3-9 combines domains of antagonistic regulators of homeotic gene complexes. EMBO J 13:3822–3831. doi:10.1002/j.1460-2075.1994.tb06693.x 7915232 PMC395295

[35] Stassen MJ, Bailey D, Nelson S, Chinwalla V, Harte PJ. 1995. The Drosophila trithorax proteins contain a novel variant of the nuclear receptor type DNA binding domain and an ancient conserved motif found in other chromosomal proteins. Mech Dev 52:209–223. doi:10.1016/0925-4773(95)00402-m 8541210

[36] Torres-Machorro AL, Clark LG, Chang CS, Pillus L. 2015. The Set3 complex antagonizes the MYST acetyltransferase Esa1 in the DNA damage response. Mol Cell Biol 35:3714–3725. doi:10.1128/MCB.00298-15 26303527 PMC4589593

[37] Kim T, Xu Z, Clauder-Münster S, Steinmetz LM, Buratowski S. 2012. Set3 HDAC mediates effects of overlapping noncoding transcription on gene induction kinetics. Cell 150:1158–1169. doi:10.1016/j.cell.2012.08.016 22959268 PMC3461055

[38] Harvey ZH, Chakravarty AK, Futia RA, Jarosz DF. 2020. A prion epigenetic switch establishes an active chromatin state. Cell 180:928–940. doi:10.1016/j.cell.2020.02.014 32109413 PMC7195540

[39] Cohen TJ, Mallory MJ, Strich R, Yao TP. 2008. Hos2P/Set3P deacetylase complex signals secretory stress through the Mpk1P cell integrity pathway. Eukaryot Cell 7:1191–1199. doi:10.1128/EC.00059-08 18487345 PMC2446675

[40] Wang A, Kurdistani SK, Grunstein M. 2002. Requirement of Hos2 histone deacetylase for gene activity in yeast. Science 298:1412–1414. doi:10.1126/science.1077790 12434058

[41] Ding SL, Liu W, Iliuk A, Ribot C, Vallet J, Tao A, Wang Y, Lebrun MH, Xu JR. 2010. The tig1 histone deacetylase complex regulates infectious growth in the rice blast fungus Magnaporthe oryzae. Plant Cell 22:2495–2508. doi:10.1105/tpc.110.074302 20675574 PMC2929099

[42] Ding S, Mehrabi R, Koten C, Kang Z, Wei Y, Seong K, Kistler HC, Xu JR. 2009. Transducin beta-like gene FTL1 is essential for pathogenesis in Fusarium graminearum. Eukaryot Cell 8:867–876. doi:10.1128/EC.00048-09 19377037 PMC2698311

[43] Hnisz D, Schwarzmüller T, Kuchler K. 2009. Transcriptional loops meet chromatin: a dual-layer network controls white-opaque switching in Candida albicans. Mol Microbiol 74:1–15. doi:10.1111/j.1365-2958.2009.06772.x 19555456 PMC2764112

[44] Hnisz D, Majer O, Frohner IE, Komnenovic V, Kuchler K. 2010. The Set3/Hos2 histone deacetylase complex attenuates cAMP/PKA signaling to regulate morphogenesis and virulence of Candida albicans. PLoS Pathog 6:e1000889. doi:10.1371/journal.ppat.1000889 20485517 PMC2869326

[45] Meng Y, Fan Y, Liao W, Lin X. 2018. Plant homeodomain genes play important roles in cryptococcal yeast-hypha transition. Appl Environ Microbiol 84:e01732-17. doi:10.1128/AEM.01732-17 PMC5930315 29500261

[46] Richter K, Haslbeck M, Buchner J. 2010. The heat shock response: life on the verge of death. Mol Cell 40:253–266. doi:10.1016/j.molcel.2010.10.006 20965420

[47] Leach MD, Stead DA, Argo E, MacCallum DM, Brown AJP. 2011. Molecular and proteomic analyses highlight the importance of ubiquitination for the stress resistance, metabolic adaptation, morphogenetic regulation and virulence of Candida albicans. Mol Microbiol 79:1574–1593. doi:10.1111/j.1365-2958.2011.07542.x 21269335 PMC3084552

[48] Hossain S, Veri AO, Cowen LE. 2020. The proteasome governs fungal morphogenesis via functional connections with Hsp90 and cAMP-protein kinase a signaling. mBio 11:e00290-20. doi:10.1128/mBio.00290-20 32317319 PMC7175089

[49] Jöhnk B, Bayram Ö, Abelmann A, Heinekamp T, Mattern DJ, Brakhage AA, Jacobsen ID, Valerius O, Braus GH. 2016. SCF ubiquitin ligase F-box protein Fbx15 controls nuclear co-repressor localization, stress response and virulence of the human pathogen Aspergillus fumigatus. PLoS Pathog 12:e1005899. doi:10.1371/journal.ppat.1005899 27649508 PMC5029927

[50] Webb JL, Ravikumar B, Atkins J, Skepper JN, Rubinsztein DC. 2003. α-synuclein is degraded by both autophagy and the proteasome. J Biol Chem 278:25009–25013. doi:10.1074/jbc.M300227200 12719433

[51] Franssens V, Bynens T, Van den Brande J, Vandermeeren K, Verduyckt M, Winderickx J. 2013. The benefits of humanized yeast models to study parkinson’s disease. Oxid Med Cell Longev 2013:760629. doi:10.1155/2013/760629 23936613 PMC3713309

[52] Franzmann T, Alberti S. 2021. Ubiquitin protein helps cells to recover from stress. Nature 597:183–184. doi:10.1038/d41586-021-02197-z 34400837

[53] Lee D, Goldberg AL. 2022. 26S proteasomes become stably activated upon heat shock when ubiquitination and protein degradation increase. Proc Natl Acad Sci U S A 119:e2122482119. doi:10.1073/pnas.2122482119 35704754 PMC9231471

[54] Wakabayashi K, Nakagawa H, Tamura A, Koshiba S, Hoshijima K, Komada M, Ishikawa T. 2007. Intramolecular disulfide bond is a critical check point determining degradative fates of ATP-binding cassette (ABC) transporter ABCG2 protein. J Biol Chem 282:27841–27846. doi:10.1074/jbc.C700133200 17686774

[55] Idnurm A, Bahn YS, Nielsen K, Lin X, Fraser JA, Heitman J. 2005. Deciphering the model pathogenic fungus Cryptococcus neoformans. Nat Rev Microbiol 3:753–764. doi:10.1038/nrmicro1245 16132036

[56] Gatchalian J, Ali M, Andrews FH, Zhang Y, Barrett AS, Kutateladze TG. 2017. Structural insight into recognition of methylated histone H3K4 by Set3. J Mol Biol 429:2066–2074. doi:10.1016/j.jmb.2016.09.020 27697561 PMC5374059

[57] Veerappan CS, Avramova Z, Moriyama EN. 2008. Evolution of SET-domain protein families in the unicellular and multicellular ascomycota fungi. BMC Evol Biol 8:190. doi:10.1186/1471-2148-8-190 18593478 PMC2474616

[58] Gong C, Xu D, Sun D, Kang J, Wang W, Xu J-R, Zhang X. 2022. FgSnt1 of the Set3 HDAC complex plays a key role in mediating the regulation of histone acetylation by the cAMP-PKA pathway in Fusarium graminearum. PLoS Genet 18:e1010510. doi:10.1371/journal.pgen.1010510 36477146 PMC9728937

[59] Marshall RS, Vierstra RD. 2019. Dynamic regulation of the 26S proteasome: from synthesis to degradation. Front Mol Biosci 6:40. doi:10.3389/fmolb.2019.00040 31231659 PMC6568242

[60] Meyer-Schwesinger C. 2019. The ubiquitin-proteasome system in kidney physiology and disease. Nat Rev Nephrol 15:393–411. doi:10.1038/s41581-019-0148-1 31036905

[61] Goldberg AL. 2003. Protein degradation and protection against misfolded or damaged proteins. Nature 426:895–899. doi:10.1038/nature02263 14685250

[62] Cao C, Xue C. 2021. More than just cleaning: ubiquitin-mediated proteolysis in fungal pathogenesis. Front Cell Infect Microbiol 11:774613. doi:10.3389/fcimb.2021.774613 34858882 PMC8631298

[63] Geddes JMH, Caza M, Croll D, Stoynov N, Foster LJ, Kronstad JW. 2016. Analysis of the protein kinase a-regulated proteome of Cryptococcus neoformans identifies a role for the ubiquitin-proteasome pathway in capsule formation. mBio 7:e01862-15. doi:10.1128/mBio.01862-15 26758180 PMC4725006

[64] Johnston HE, Samant RS. 2021. Alternative systems for misfolded protein clearance: life beyond the proteasome. FEBS J 288:4464–4487. doi:10.1111/febs.15617 33135311

[65] Qu J, Ren X, Xue F, He Y, Zhang R, Zheng Y, Huang H, Wang W, Zhang J. 2020. Specific knockdown of α-synuclein by peptide-directed proteasome degradation rescued its associated neurotoxicity. Cell Chem Biol 27:751–762. doi:10.1016/j.chembiol.2020.03.010 32359427

[66] Geiler-Samerotte KA, Dion MF, Budnik BA, Wang SM, Hartl DL, Drummond DA. 2011. Misfolded proteins impose a dosage-dependent fitness cost and trigger a cytosolic unfolded protein response in yeast. Proc Natl Acad Sci U S A 108:680–685. doi:10.1073/pnas.1017570108 21187411 PMC3021021

[67] Parag HA, Raboy B, Kulka RG. 1987. Effect of heat shock on protein degradation in mammalian cells: involvement of the ubiquitin system. EMBO J 6:55–61. doi:10.1002/j.1460-2075.1987.tb04718.x 3034579 PMC553356

[68] Li XM, Chao DY, Wu Y, Huang X, Chen K, Cui LG, Su L, Ye WW, Chen H, Chen HC, Dong NQ, Guo T, Shi M, Feng Q, Zhang P, Han B, Shan JX, Gao JP, Lin HX. 2015. Natural alleles of a proteasome α2 subunit gene contribute to thermotolerance and adaptation of African rice. Nat Genet 47:827–833. doi:10.1038/ng.3305 25985140

[69] Panek J, Gang SS, Reddy KC, Luallen RJ, Fulzele A, Bennett EJ, Troemel ER. 2020. A cullin-RING ubiquitin ligase promotes thermotolerance as part of the intracellular pathogen response in Caenorhabditis elegans. Proc Natl Acad Sci U S A 117:7950–7960. doi:10.1073/pnas.1918417117 32193347 PMC7148578

[70] Liu Y, Xiao S, Sun H, Pei L, Liu Y, Peng L, Gao X, Liu Y, Wang J. 2020. AtPPRT1, an E3 ubiquitin ligase, enhances the thermotolerance in arabidopsis. Plants 9:1074. doi:10.3390/plants9091074 32825569 PMC7569766

[71] Kuckelkorn U, Knuehl C, Boes-Fabian B, Drung I, Kloetzel PM. 2000. The effect of heat shock on 20S/26S proteasomes. Biol Chem 381:1017–1023. doi:10.1515/BC.2000.125 11076035

[72] Liu OW, Chun CD, Chow ED, Chen C, Madhani HD, Noble SM. 2008. Systematic genetic analysis of virulence in the human fungal pathogen Cryptococcus neoformans. Cell 135:174–188. doi:10.1016/j.cell.2008.07.046 18854164 PMC2628477

[73] Toffaletti DL, Rude TH, Johnston SA, Durack DT, Perfect JR. 1993. Gene transfer in Cryptococcus neoformans by use of biolistic delivery of DNA. J Bacteriol 175:1405–1411. doi:10.1128/jb.175.5.1405-1411.1993 8444802 PMC193227

