# Supplementary material for: Unraveling the mechanism of thermotolerance by Set302 in Cryptococcus neoformans
Source: Microbiol Spectr. 2024 Jun 14;12(8):e04202-23. doi: 10.1128/spectrum.04202-23 (PMC11302353; doi:10.1128/spectrum.04202-23)
Supplement: Supplemental figures — Fig. S1-S4. [file spectrum.04202-23-s0001.docx]

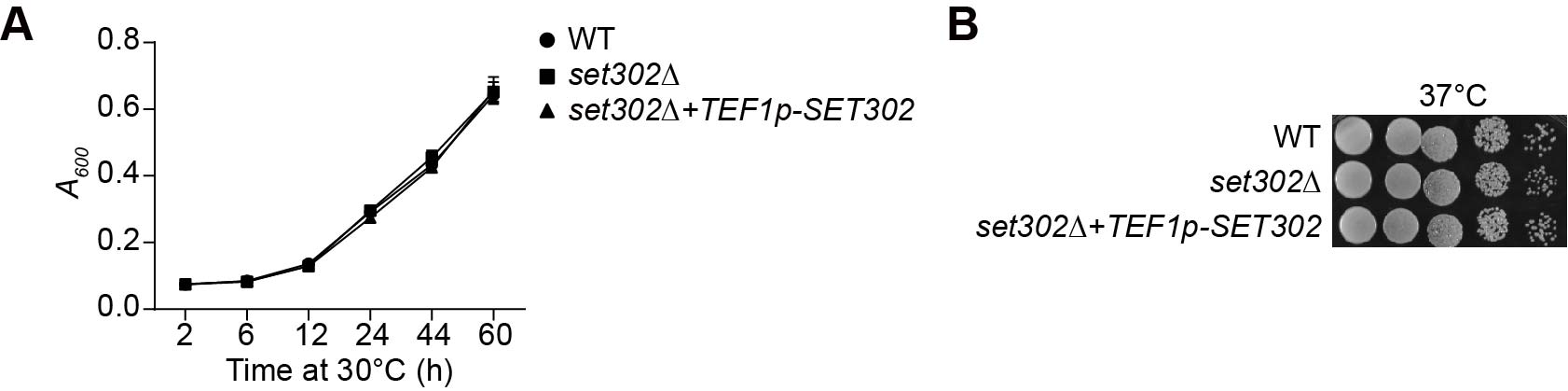


Figure S1 A. Growth curve of fungal cells in liquid cultures. Wild-type, *set302∆* and *set302∆+**TEF1p-SET302* strains were grown in YPD liquid medium at 30°C. Cell growths (absorbance at 600 nm) were measured at indicated time points. B. Spot dilution assays with wild-type, *set302∆* and *set302∆+TEF1p-SET302* strains were performed on YPD agar and incubated at 37°C for two days.


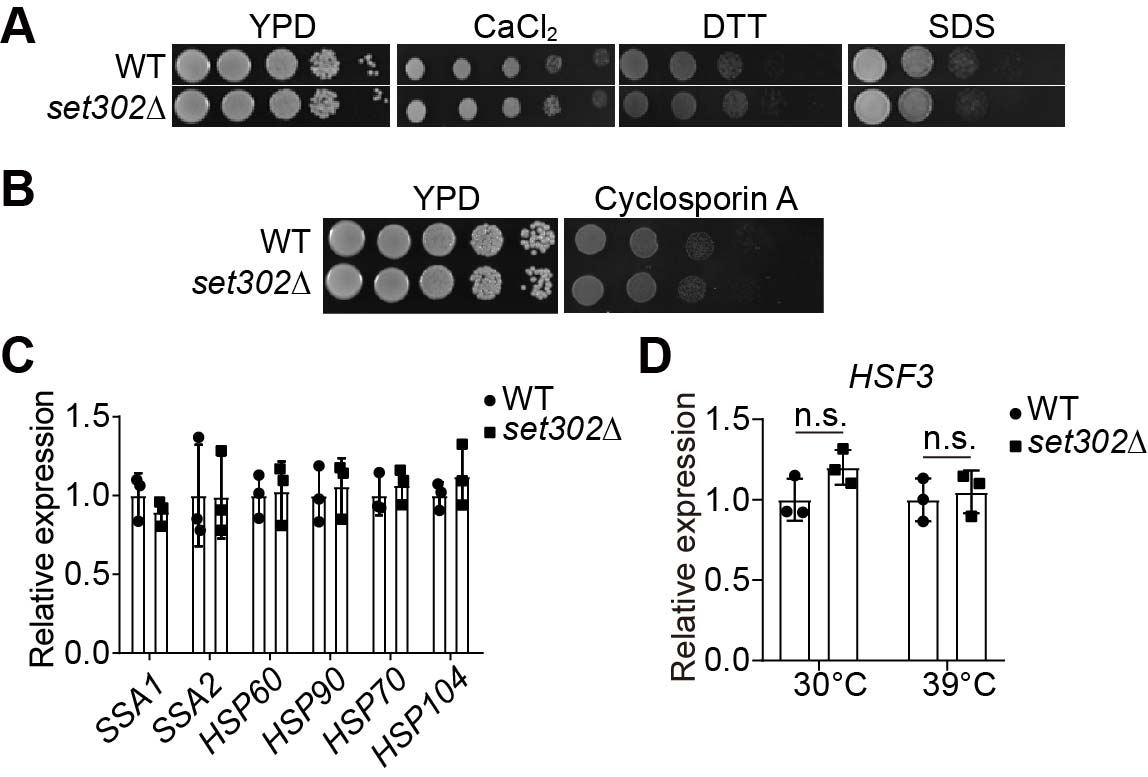


Figure S2 A. Spot dilution assays with wild-type and *set302∆* strains were performed on YPD agar with CaCl_2_, DTT or SDS, and then incubated at 30°C for two days. B. Spot dilution assays with wild-type and *set302∆* strains were performed on YPD agar with cyclosporin A, a calcineurin inhibitor, and incubated at 30°C for two days. C. Quantitative RT-PCR was performed using total RNA from wild-type and *set302∆* strains at 39°C and the gene expression of representative HSPs was measured. D. Quantitative RT-PCR was performed using total RNA from wild-type and *set302∆* strains at 30°C and 39°C and the gene expression of *HSF3* was measured.


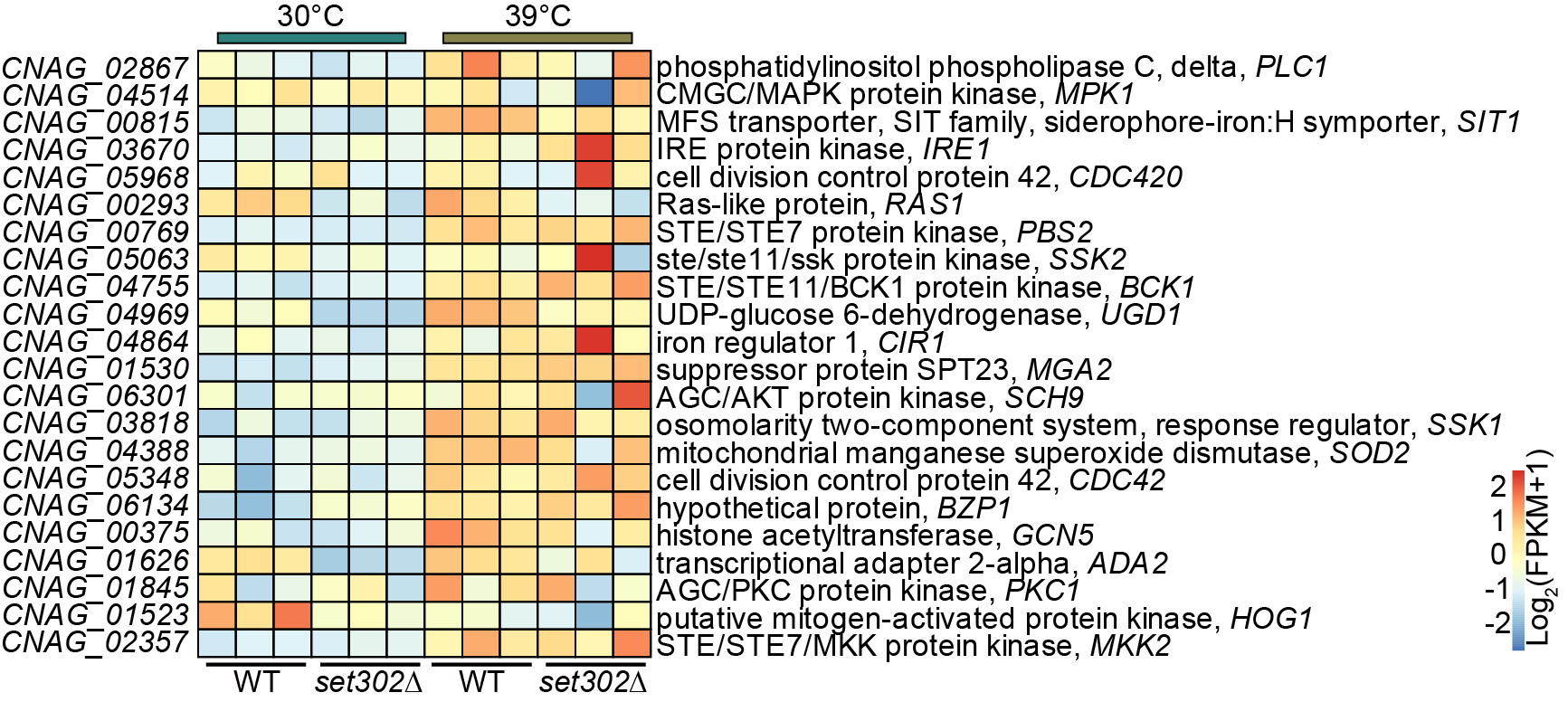


Figure S3 Heatmap of thermotolerance-related genes from transcriptome. These thermotolerance-related genes were gained from the CryptoNet database (<https://www.inetbio.org/cryptonet/>). The heatmap analysis is shown as normalized genes expression (FPKM).


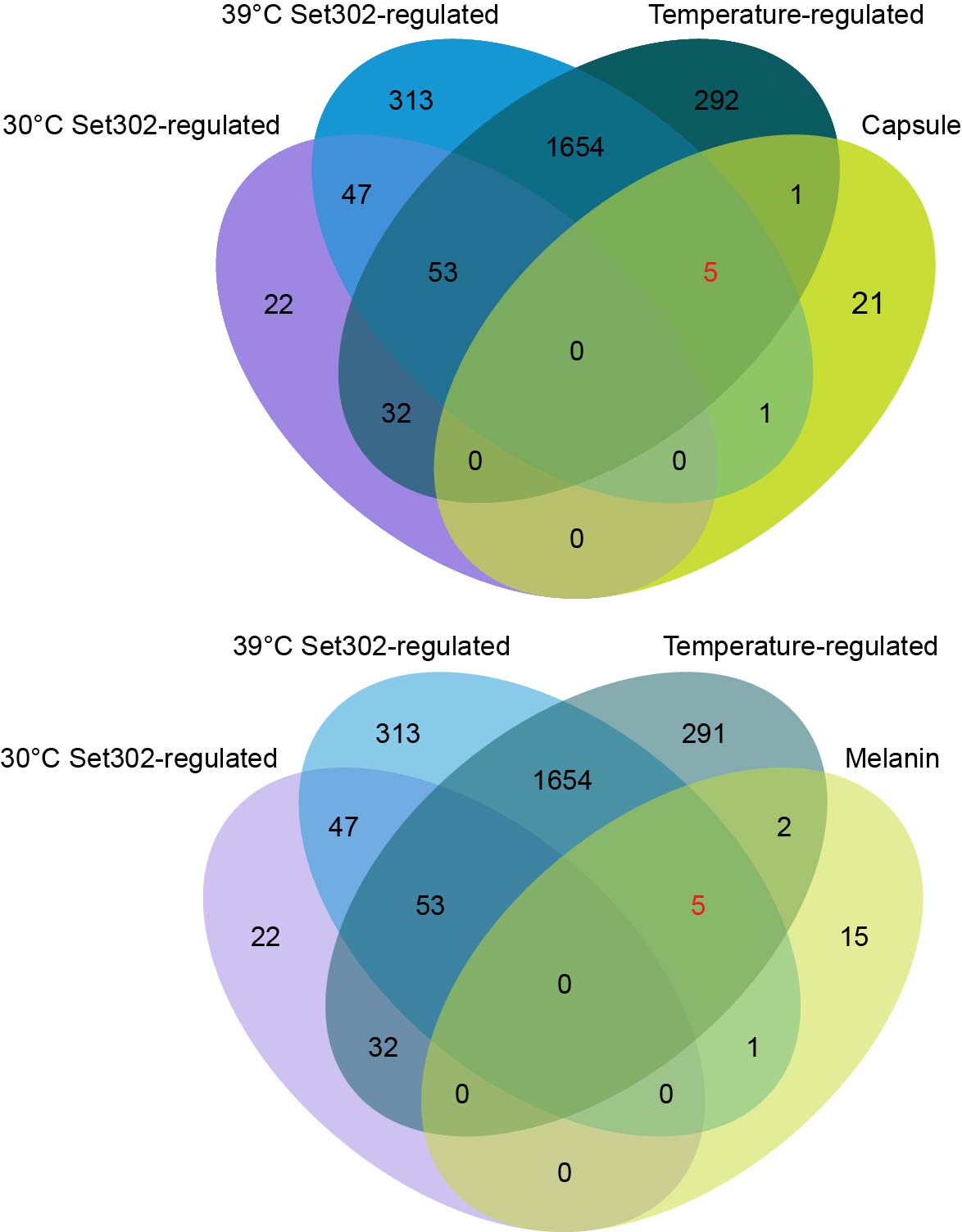


Figure S4 Venn diagrams depict the comparison of the number of genes from different groups. 30°C Set302-regulated genes group and 39°C Set302-regulated genes group correspond to genes with changed expression when Set302 is deleted at 30°C or 39°C, respectively. Temperature-regulated genes group corresponds to the genes with changed expression when the temperature is shifted from 30°C to 39°C. Capsule and melanin genes are gained from CryptoNet database.
